# Maternal nutrition induces gene expression changes in fetal muscle and adipose tissues in sheep

**DOI:** 10.1186/1471-2164-15-1034

**Published:** 2014-11-28

**Authors:** Francisco Peñagaricano, Xin Wang, Guilherme JM Rosa, Amy E Radunz, Hasan Khatib

**Affiliations:** Department of Animal Sciences, University of Wisconsin-Madison, 1675 Observatory Drive, Madison, WI 53706 USA; College of Animal Science and Technology, Northwest A&F University, Yangling, Shaanxi 712100 China; Department of Animal and Food Science, University of Wisconsin-River Falls, River Falls, WI 54022 USA

**Keywords:** Maternal nutrition, Transcriptome analysis, RNA-sequencing, Fetal programming, Sheep

## Abstract

**Background:**

Maternal nutrition during different stages of pregnancy can induce significant changes in the structure, physiology, and metabolism of the offspring. These changes could have important implications on food animal production especially if these perturbations impact muscle and adipose tissue development. Here, we evaluated the impact of different maternal isoenergetic diets, alfalfa haylage (HY; fiber), corn (CN; starch), and dried corn distillers grains (DG; fiber plus protein plus fat), on the transcriptome of fetal muscle and adipose tissues in sheep.

**Results:**

Prepartum diets were associated with notable gene expression changes in fetal tissues. In longissimus dorsi muscle, a total of 224 and 823 genes showed differential expression (FDR ≤0.05) in fetuses derived from DG vs. CN and HY vs. CN maternal diets, respectively. Several of these significant genes affected myogenesis and muscle differentiation. In subcutaneous and perirenal adipose tissues, 745 and 208 genes were differentially expressed (FDR ≤0.05), respectively, between CN and DG diets. Many of these genes are involved in adipogenesis, lipogenesis, and adipose tissue development. Pathway analysis revealed that several GO terms and KEGG pathways were enriched (FDR ≤0.05) with differentially expressed genes associated with tissue and organ development, chromatin biology, and different metabolic processes.

**Conclusions:**

These findings provide evidence that maternal nutrition during pregnancy can alter the programming of fetal muscle and fat tissues in sheep. The ramifications of the observed gene expression changes, in terms of postnatal growth, body composition, and meat quality of the offspring, warrant future investigation.

**Electronic supplementary material:**

The online version of this article (doi:10.1186/1471-2164-15-1034) contains supplementary material, which is available to authorized users.

## Background

Fetal developmental programming describes the process whereby a maternal stimulus or insult during a critical period of development has lasting or lifelong consequences [1]. Maternal nutrition is considered a major intrauterine environmental factor in fetal development. In fact, it is well established that maternal nutrition during different stages of pregnancy can induce permanent changes in the structure, physiology, and metabolism of the offspring [2, 3]. Epidemiological studies in humans have shown that nutrient restriction during gestation increases the incidence of postnatal disorders, including obesity and coronary heart disease [4, 5]. In addition, experimental studies using rodent models have shown that both maternal undernutrition and overnutrition have significant effects on fetal development with long term implications. For instance, maternal dietary restriction leads to delayed postnatal growth and elevated blood pressure of offspring [6]. Restriction of maternal protein intake throughout gestation resulted in decreased birth weight, perturbations to renin-angiotensin homeostasis, kidney disorders, and adult hypertension [7]. Maternal high-fat over-feeding during pregnancy resulted in several disorders in the offspring, including abnormal glucose homeostasis, abnormal serum lipid profiles, and increased adiposity [8]. Overall, these studies are consistent with the idea that maternal nutrition during pregnancy can induce remarkable effects on fetal development, which in turn may predispose the offspring to metabolic, endocrine, and cardiovascular disorders in postnatal life.

Postnatal effects of maternal nutrition on growth, productivity, and reproduction of the offspring could have important implications in the livestock industry. Indeed, evidence suggests that maternal nutrient intake can alter subsequent growth, skeletal muscle development, body composition, and energy metabolism in early postnatal life of offspring in livestock species [9, 10]. Different prepartum maternal energy sources fed during late gestation in beef cattle resulted in changes in birth weight and postnatal muscle and fat deposition in progeny [11–14]. Similarly, prepartum diets during mid to late gestation in sheep had significant effects on postnatal carcass composition in terms of both fat and muscle deposition [15]. Dietary components, such as protein content or fat supplementation, have been associated with alterations in the offspring performance. Maternal protein supplementation during late gestation has been positively associated with postnatal growth and adipose deposition in steers (male progeny), and also postweaning body weight and fertility in heifers (female progeny) in beef cattle [16, 17]. Fat supplementation in gestating ewes increased cold tolerance and improved survival of their lambs [18]. Maternal energy sources, such as starch or fiber, may also impact fetal development and subsequent performance of the offspring. Maternal starch-based diets in cattle have been associated with greater calf birth weights compared with fiber-based prepartum diets [19, 20]. Therefore, these studies in livestock species clearly show that prepartum diets can affect the performance of the offspring, including postnatal growth, body composition, and carcass weight and may have significant implications in food animal production.

Fetal programming of skeletal muscle and adipose tissue development may have substantial effects on economic viability of livestock enterprises through impacts on efficiency of production and product quality. Skeletal muscle mass is largely determined by the number and the size of muscle fibers. Muscle fibers are formed exclusively during the prenatal stage, especially from early to mid-gestation, and there is no further net increase in the number of these fibers after birth [21]. In contrast, the size of muscle fibers depends on satellite cells which proliferate, synthesize myofibrillar proteins, and fuse with existing muscle fibers to increase muscle fiber size which starts to occur in late gestation and continues in postnatal development [21]. Therefore, programming of muscle tissue during fetal development which could lead to a lesser number and smaller size of muscle fibers would reduce muscle mass and have negative effects on efficiency of animal production [21, 22]. Studies in sheep have shown that maternal undernutrition in early gestation mainly affects fetal muscle fiber number while inadequate nutrition during late gestation impacts fetal muscle fiber size [23–25]. The majority of fetal adipose tissue is developed during the final third of gestation, and it is now well established that maternal nutrition plays a key role in its development [26]. In fact, both maternal undernutrition and overnutrition can result in the long-term programming of adipose tissue abundance and function [27, 28]. Ovine studies have shown that maternal nutrient restriction during early to mid-gestation, coincident with the period of maximal placental growth, results in increased perirenal adipose tissues at birth [29]. Conversely, maternal undernutrition during late gestation, which is coincident with the period of maximal fetal growth, results in reduced adiposity in the offspring [29]. Furthermore, overnutrition during late pregnancy induces overexpression of genes regulating adipogenesis and lipogenesis in fetal perirenal fat tissue in sheep, which in turn may result in increased adiposity in later life [30]. These studies show that maternal nutrition can alter the composition and abundance of fetal skeletal muscle and fat depots that may affect livestock production.

Previous studies have reported the effects of maternal nutrition on fetal development in livestock species, including fetal programming of muscle and fat tissues, using extreme undernutrition or overnutrition conditions. Few studies have evaluated the possible impact of subtle differences in maternal diets, such as different energy sources or subtle differences in protein content, on the overall expression of the fetal genome. Maternal diets containing different energy sources, such as starch, fiber or fat, or showing subtle differences in protein content, may alter fetal development in livestock species to have long term implications in food animal production. We have previously reported that maternal diets differing in fat and protein content alter postnatal growth traits in cattle and sheep [11, 15, 31]. The mechanisms, however, by which these diets affect muscle and fat tissues are not known. Additionally, our recent study has shown that the expression of imprinted genes in fetal tissues was influenced by maternal diets that differed in energy sources [32], but genome-wide expression differences have not yet been explored. As such, the objective of this study was to investigate the impact of different maternal dietary feed sources during mid-to-late gestation on the transcriptome of fetal muscle and adipose tissues in sheep.

## Methods

### Ethics statement

The College of Agriculture and Life Sciences Animal Care and Use Committee of the University of Wisconsin-Madison approved the procedures used in this study.

### Animals, experimental design, and maternal diets

Multiparous Polypay ewes were used in a completely randomized design to evaluate the effect of different maternal diets during mid-to-late gestation on the transcriptome of different fetal tissues. Ewes were naturally bred to a single sire and from days 67 ± 3 of gestation until necropsy (days 130 ± 1), they were individually fed one of three diets where the primary energy source was alfalfa haylage (HY; fiber), corn (CN; starch), or dried corn distillers grains (DG; fiber, protein and fat). These diets were formulated to meet nutritional requirements of the ewes during these stages of production. Table 1 shows the daily nutrient intake of the ewes by dietary treatment during the experiment. Intake of CN and DG diets was limited to achieve isoenergetic intake among dietary treatments relative to an ad libitum intake of HY, and intake of CN and DG diets was adjusted to achieve similar body weight gains of ewes from days 67 to days 130 of gestation.

**Table 1:** **Daily nutrient intake of ewes from days 67 to 130 of gestation on a dry matter basis**

	Diets		
Item	HY	CN	DG
DMI, kg/d	2.03	1.17	1.18
Alfalfa haylage	2.03	0.14	0.17
Corn	-	0.80	-
DDGS	-	-	0.77
Supplement	-	0.23	0.24
***Nutrient intake***			
Crude protein (g/d)	383.26	130.63	309.84
NDF (g/d)	940.10	198.82	508.16
Crude fat (g/d)	85.97	84.94	114.02

Diets fed to dams: **HY** = ad libitum fed alfalfa haylage; **CN** = limit-fed whole shell corn;

**DG** = limit-fed corn dried distiller’s grains (adapted from Lan et al. [32]).

### Tissue collection

Ewes were necropsied on day 130 ± 1 of gestation. A total of 26 fetuses were removed from 15 dams. From the fetuses three different tissues were collected including one muscle, longissimus dorsi muscle from the left side of the fetus, and two adipose tissues, perirenal adipose depot and subcutaneous adipose depot near the shoulder blade. These tissues were immediately frozen at -80°C until RNA extraction was performed.

### RNA extraction and preparation of pooled samples

Total RNA was extracted from tissue samples using the RNeasy Mini kit (QIAGEN, Valencia, CA, USA) and then treated with DNase-free DNase Set (QIAGEN) to avoid genomic DNA amplification. Concentrations and OD_260/280_ ratios of RNA samples were measured with the Nanodrop ND-1000 spectrophotometer (Nanodrop Technologies, Montchanin, DE).

The RNA samples from the 26 fetuses were pooled to generate four biological replicates per maternal diet and tissue. In particular, per each diet and tissue, two RNA pools were created from male fetuses and two RNA pools were created from female fetuses. The description of the pools including number of fetuses and dams can be found in Additional file 1.

### Library generation and RNA sequencing

A total of 50 ng of RNA from each pool was used to prepare sequencing libraries following Illumina’s mRNA-Seq protocol. Libraries were sequenced with Illumina’s HiSeq 2000 at the Biotechnology Center in the University of Wisconsin-Madison. The 36 libraries (i.e., 12 libraries per tissue, three tissues in total) were barcoded, multiplexed, and then sequenced. A read was defined as a 100 bp cDNA fragment sequenced from a single end. Approximately 30 million reads were sequenced from each library.

### Mapping reads to the reference genome

Raw sequence reads were mapped directly to the reference sheep genome (Oar_v3.1) using the software package Tophat (v2.0.4) [33]. A two-step approach was used in order to maximize sensitivity to splice junction discovery: the first alignment was performed in each of the samples independently, and then novel splice junctions plus known splice junctions from Ensembl annotation were combined and supplied to Tophat for a second alignment. This approach allows for full utilization of the novel junctions identified in the samples. A maximum of two mismatches were allowed and reads that mapped equally well to more than 40 genomic locations were discarded.

### Assembly of transcripts and estimation of abundance

The resulting alignments were used to reconstruct transcript models using the software package Cufflinks (v2.1.1) [34]. This program constructs a parsimonious set of transcript models that best explain the read alignments observed in the samples. In addition, the tool cuffmerge was used for merging together each of the assemblies with the reference sheep annotation file in order to combine novel transcripts with known annotated transcripts. This strategy maximizes the overall quality of the final assembly. Finally, abundances of transcripts were scaled via the median of the geometric means of fragment counts across all libraries (as described in [35]) and corrected for sequence bias [36] in order to improve expression estimates.

### Overall gene expression analysis

Differentially expressed genes were detected using Cuffdiff, a companion tool of Cufflinks. Cuffdiff performs differential analysis at both gene and transcript-level resolution and controls for both variability across replicates and uncertainty in abundance expression estimates caused by ambiguously mapped reads [37]. In addition, Cuffdiff controls for cross-replicate variability and also read-mapping ambiguity by using a model for fragment counts based on the beta negative binomial distribution [37]. Here, the analyses were performed using the default settings for all parameters. Finally, to account for multiple hypothesis testing and control the false discovery rate, *P*-values reported by Cuffdiff were corrected using the Benjamini-Hochberg procedure [38].

### Validation of differentially expressed genes

Four genes that showed significant differential expression between CN and DG in subcutaneous adipose depot tissue were chosen for validation of the RNA-Seq results: ankyrin repeat domain 1 (*ANKRD1*, also known as *CARP*), kringle containing transmembrane protein 1 (*KREMEN1*), muscle-related coiled-coil protein (*MURC*), and synaptopodin 2-like (*SYNPO2L*). The RNA samples used for RNA-Seq were used here for validation of differential expression. A total of 1 μg RNA from each sample was used to synthesize cDNA using the iScript cDNA Synthesis Kit (Bio-Rad Laboratories, Hercules, CA, USA) following the manufacturer’s instructions. Quantitative real-time PCR (qRT-PCR) was used to estimate the fold change in expression between treatments. The ribosomal protein L19 (RPL19) gene was chosen as internal control because of its stable expression across samples. All samples were run in triplicates in the ECO real-time PCR system (Illumina, San Diego, CA) using iQSYBR Green Supermix (Bio-Rad Laboratories, CA). Primers were designed to cross exon-exon junctions to minimize the potential of amplifying genomic DNA and are shown in Table 2. The relative gene expression values were calculated using the 2^-ΔΔCt^ method [39].

**Table 2:** **Primers used for the validation of gene expression**

Gene	Primer sequence 5’ → 3’	Amplicon size (bp)
*ANKRD1*	F: GTATCTCCTTCCGGTCTTTGG	117
	R: CGCGATAATTGCTCAGCAC	
*KREMEN1*	F: GATGGCAGGGTCATCATCTTC	67
	R: GAGGTCATGGCCGAGTAATTC	
*MURC*	F: CCTCTGGACTCTCAATCAAGC	93
	R: AACAAATTCCGTGTGGTCAT	
*PRL19*	F: CAACTCCCGCCAGCAGAT	76
	R: CCGGGAATGGACAGTCACA	
*SYNPO2L*	F: CTGTGCGACCGCCACCCT	115
	R: GTGTTTCAGGGATTCCAGC	

### Gene set enrichment analysis

The significant enrichment of Gene Ontology (GO) terms and KEGG pathways with genes differentially expressed between maternal diets was analyzed using Fisher’s exact test, a test of proportions based on the cumulative hypergeometric distribution [40]. For each comparison of interest, genes that showed a FDR ≤0.20 and had ENSEMBL annotations were tested against the background set of all genes with ENSEMBL annotations. The procedure proposed by Benjamini and Hochberg [38] was applied in order to account for multiple testing. Functional categories with FDR ≤0.05 were considered significant. These analyses were performed using the procedure FatiGO [41], implemented on the platform Babelomics [42], and using the Bioconductor packages *org.Bt.eg.db*, *GO.db*, and *KEGG.db* that are available in the R language/environment.

## Results

### Sequencing of the transcriptome of different fetal sheep tissues

To assess the effects of three different maternal diets on the transcriptome of three different fetal tissues in sheep, a total of 36 pooled samples (12 pooled samples per tissue with 4 biological replicates per diet) were analyzed using RNA-sequencing. Additional file 2 shows the results of the alignment of the sequencing reads to the sheep reference genome. Approximately, 30 million reads were sequenced for each sample. Sequencing reads were aligned against the recent ovine reference genome (Oar_v3.1) using the software package Tophat. On average, 83% of the total reads were successfully mapped by allowing no more than two mismatches and restricting the alignments to at most 40 genomic locations (Additional file 2). Among the aligned reads, approximately 86% and 65% were mapped to unique genomic regions in muscle and adipose tissues, respectively. Sequencing data can be accessed by GEO with the accession number GSE62938.

### Differential gene expression in longissimus dorsi muscle tissue

**Figure 1:**
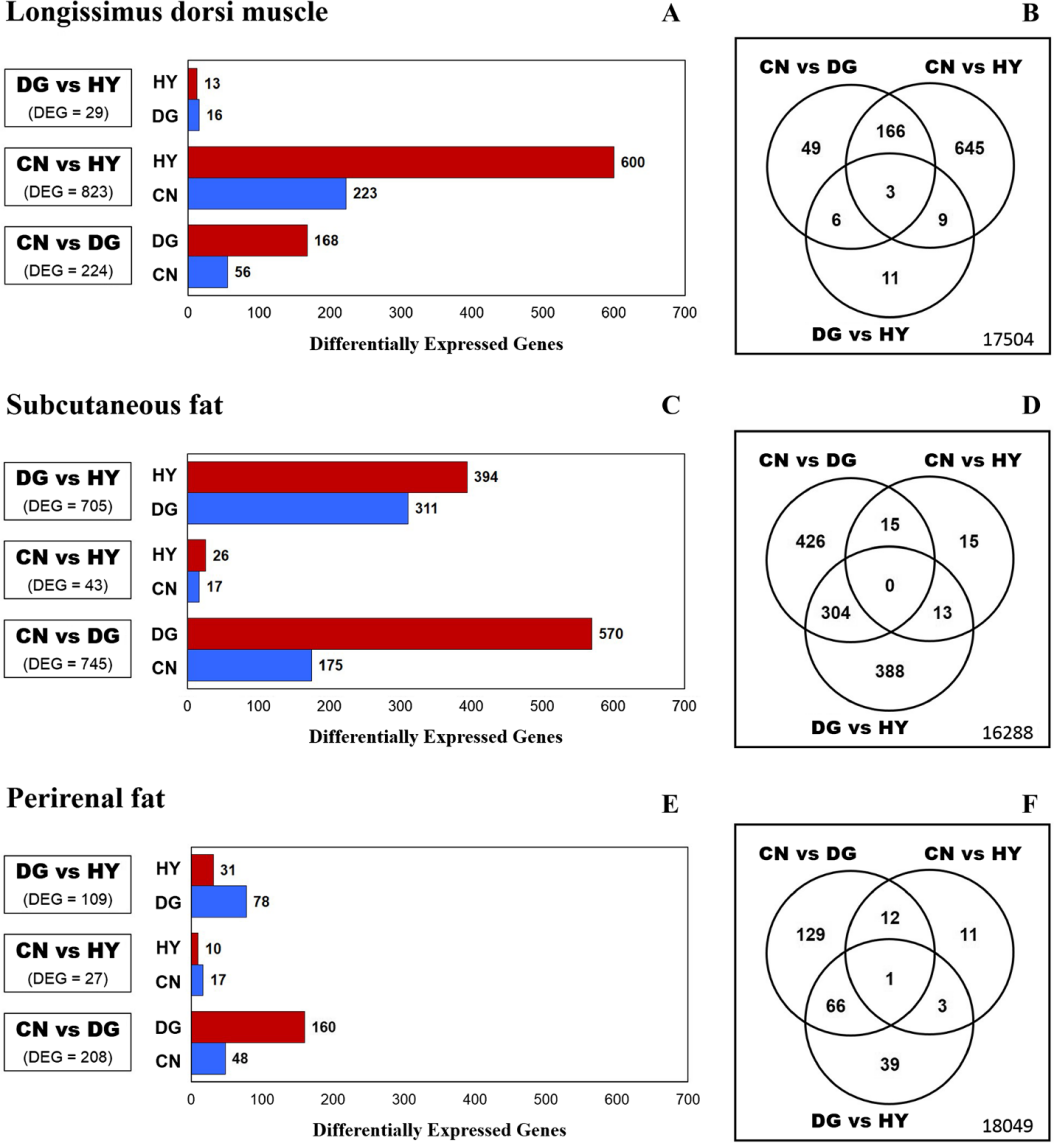
**Comparison of overall gene expression between maternal diets in three different fetal tissues.** Fetal tissues: *longissimus dorsi* **(A-B)**, *subcutaneous adipose depot* **(C-D)**, and *perirenal adipose depot* **(E-F)**. Diets fed to dams: **HY** = ad libitum fed alfalfa haylage; **CN** = limit-fed whole shell corn; **DG** = limit-fed corn dried distillers grains. The bar graphs show the number of genes up-regulated in each diet for each of the three pairwise comparisons. The Venn diagrams show the overlap between genes that showed significant differential expression in each of the three pairwise comparisons.

A total of 18,393 genes were tested for differential expression between maternal diets in fetal longissimus dorsi muscle tissue. Marked differences in gene expression were found between CN diet and the other two maternal diets. Controlling FDR at 0.05, a total of 224, 823 and 29 genes showed differential expression between CN vs. DG, CN vs. HY, and DG vs. HY, respectively (Figure 1A). Notably, the expression of most of these genes was decreased by maternal CN diet. In particular, 168 out of 224 genes, and 600 out of 823 genes showed higher expression in fetuses from DG and HY compared with those derived from CN diet, respectively (Figure 1A). Maternal diets DG and HY induced a very similar pattern of gene expression in fetal muscle; indeed, only 29 genes showed differential expression at a FDR ≤0.05 between these two diets and none of them were significant at a FDR ≤0.01.

Figure 1B shows the overlap between genes that were differentially expressed in each of the three pairwise comparisons. Interestingly, a total of 166 genes differed simultaneously between CN diet and the other two maternal diets. Many of these genes are directly involved in embryonic and fetal development [e.g., ankyrin repeat domain 11 (*ANKRD11*), axin 1 (*AXIN1*), epsin 1 (*EPN1*), epsin 2 (*EPN2*), guanine nucleotide binding protein (G protein) alpha 12 (*GNA12*), methyl-CpG binding domain protein 3 (*MBD3*), WD and tetratricopeptide repeats 1 (*WDTC1*)], skeletal muscle cell and tissue differentiation [e.g., ankyrin repeat domain 1 (*ANKRD1*), B cell CLL/lymphoma 9-like (*BCL9L*), histone cell cycle regulator (*HIRA*), myogenic differentiation 1 (*MYOD1*), sarcoglycan alpha (*SGCA*), tripartite motif containing 72, E3 ubiquitin protein ligase (*TRIM72*)], and muscle myosin complex and sarcomere organization [e.g., myosin, heavy chain 13, skeletal muscle (*MYH13*), nebulin-related anchoring protein (*NRAP*), parvin beta (*PARVB*), synaptopodin 2-like (*SYNPO2L*), titin-cap (*TCAP*)]. Importantly, the expression of all these genes was decreased in fetuses derived from dams fed CN diet (FDR ≤0.05).

To gain insight into the biological processes that could be regulated differentially between maternal diets, we performed a gene set enrichment analysis. Many functional categories that were significantly enriched (FDR ≤0.05) with differentially expressed genes were detected simultaneously in both CN vs. DG and CN vs. HY pairwise comparisons. In particular, GO terms *structural constituent of ribosome*, *ribosome*, *translation*, *chromatin*, and *nucleosome assembly*, and KEGG pathway *ribosome* were all significantly enriched (FDR ≤0.05) with genes differentially expressed in fetal muscle between CN and the other two maternal diets. We further characterized two relevant GO terms, *chromatin* and *ribosome*, in order to assess the general pattern of gene expression within these pathways. In the case of the GO term *chromatin*, the expression of eight of the nine significant genes was decreased in fetuses from CN-fed dams. On the other hand, 26 out of 36 differentially expressed genes in the GO term *ribosome* were up-regulated in the muscle of fetuses derived from CN dietary treatment. Moreover, seven different KEGG pathways closely related to energetic metabolism, including *glycolysis and gluconeogenesis*, *citrate cycle*, *valine, leucine and isoleucine degradation*, *propanoate metabolism*, and *insulin signaling pathway*, showed significant enrichment (FDR ≤0.05) of differentially expressed genes between CN and HY diets (Table 3). Notably, the expression of most genes within these pathways was decreased by maternal CN diet (Table 3).

**Table 3:** **KEGG pathways significantly enriched with differentially expressed genes (DEG) between CN and HY maternal diets in fetal longissimus dorsi muscle tissue**

KEGG term (ID)	No. genes	No. DEG (↑ HY)	***q*** -value
Glycolysis/Gluconeogenesis (00010)	32	10 (9)	0.050
Citrate cycle (TCA cycle) (00020)	23	10 (10)	0.018
Galactose metabolism (00052)	18	7 (7)	0.050
Valine, (iso)leucine degradation (00280)	32	14 (13)	0.001
Starch and sucrose metabolism (00500)	19	8 (8)	0.035
Propanoate metabolism (00640)	22	9 (8)	0.022
Ribosome (03010)	48	22 (3)	< 0.001
Insulin signaling pathway (04910)	104	25 (23)	0.035

Diets fed to dams: **CN** = limit-fed whole shell corn; HY = ad libitum fed alfalfa haylage.

### Differential gene expression in subcutaneous adipose tissue

Noticeable differences in gene expression in fetal subcutaneous adipose tissue were found between DG maternal dietary feed source and the other two maternal diets. Of the 17,449 genes evaluated for differential expression, a total of 745, 705 and 43 genes showed differential expression (FDR ≤0.05) between DG vs. CN, DG vs. HY, and CN vs. HY, respectively (Figure 1C). Most of the significant genes showed higher expression in fetuses derived from DG-fed dams compared with CN-fed dams, while similar proportion of highly and lowly expressed genes was found in the comparison between DG and HY diets (Figure 1C). Interestingly, only 43 genes showed differential expression at a FDR ≤0.05 between CN and HY dietary treatments indicating very similar pattern of overall gene expression (Figure 1C). None of these 43 genes was significant at a FDR ≤0.01.

Among the differentially-expressed genes (FDR ≤0.05) in DG vs. CN and DG vs. HY, a total of 304 genes were detected in both pairwise comparisons (Figure 1D). Many of these genes are closely related to embryonic and fetal development [e.g., angiomotin (*AMOT*), integrin, beta 6 (*ITGB6*), shisa family member 2 (*SHISA2*), SRY (sex determining region Y)-box 6 (*SOX6*), transforming growth factor, beta 2 (*TGFB2*), ubiquitin protein ligase E3 component n-recognin 3 (*UBR3*), WD and tetratricopeptide repeats 1 (*WDTC1*)], adipose tissue development [e.g., acetoacetyl-CoA synthetase (*AACS*), AT rich interactive domain 5B (MRF1-like) (*ARID5B*), patatin-like phospholipase domain containing 3 (*PNPLA3*)], cholesterol and fatty acid biosynthetic process [e.g., MLX interacting protein-like (*MLXIPL*), protein kinase, AMP-activated, alpha 2 catalytic subunit (*PRKAA2*), protein kinase, AMP-activated, beta 2 non-catalytic subunit (*PRKAB2*)], and metabolism of lipids and lipoproteins [e.g., angiopoietin-like 4 (*ANGPTL4*), cell death-inducing DFFA-like effector A (*CIDEA*), cell death-inducing DFFA-like effector C (*CIDEC*), carnitine palmitoyltransferase 1A (*CPT1A*), nuclear receptor subfamily 1, group D, member 2 (*NR1D2*), phospholipase A2, group VII (platelet-activating factor acetylhydrolase, plasma) (*PLA2G7*)]. The majority of these genes showed higher expression in the fetuses derived from dams fed DG diet.

**Figure 2:**
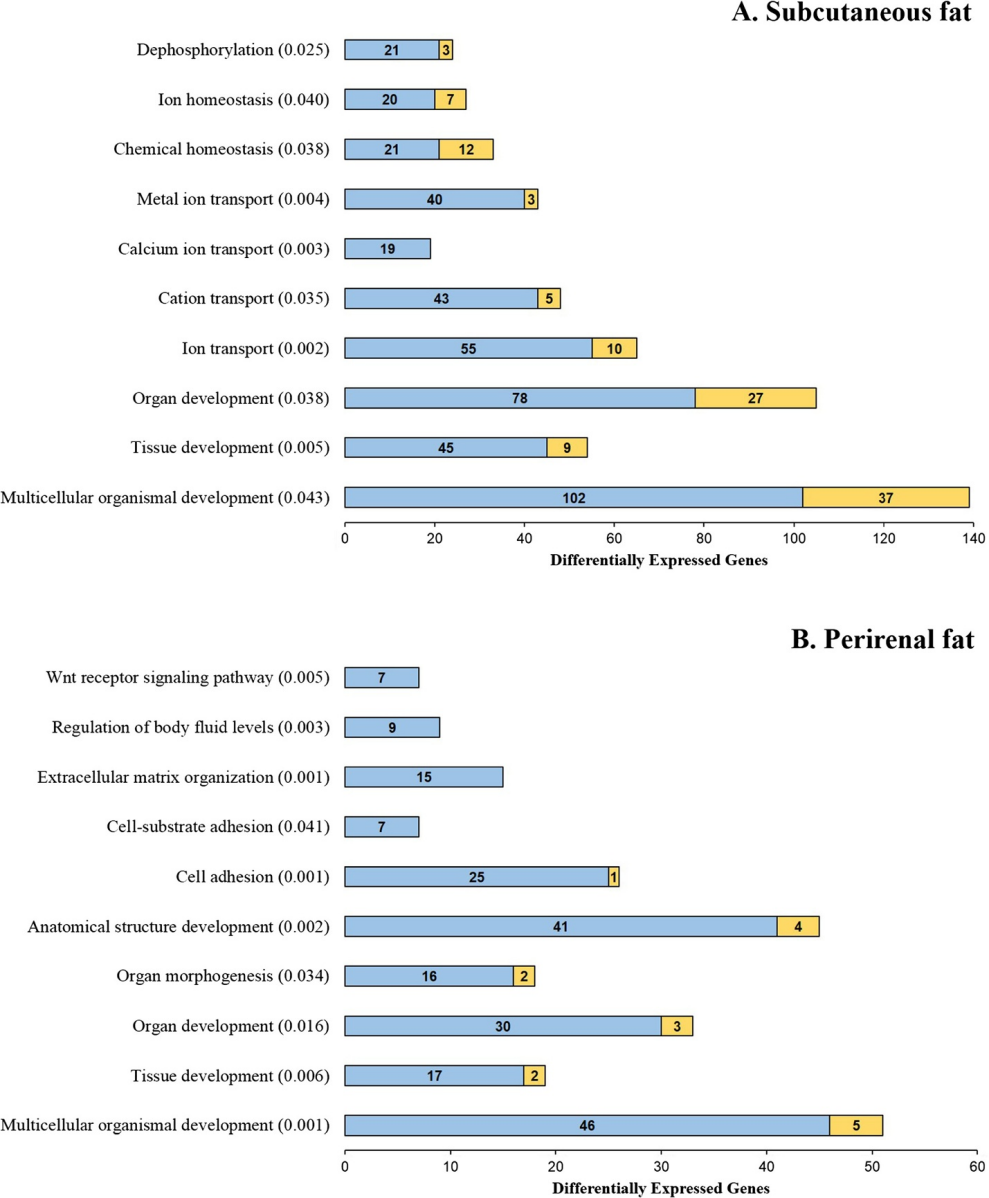
**Gene Ontology biological process terms significantly enriched with differentially expressed genes between DG and CN maternal diets in fetal subcutaneous adipose tissue (A) and fetal perirenal adipose tissue (B).** The graphs show the number of differentially expressed genes per each significant functional category (*q*-values). Genes up-regulated in DG diet are in blue while genes up-regulated in CN diet are in yellow.

Gene set enrichment analysis was performed to further characterize the processes that could be regulated differentially in fetal subcutaneous fat due to maternal nutrition. Interestingly, we found three functional categories, GO terms *dephosphorylation*, *phosphatase activity*, and *phosphoric ester hydrolase activity* that were enriched (FDR ≤0.05) with significant genes detected in both pairwise comparisons involving DG maternal diet, i.e., DG vs. CN and DG vs. HY comparisons (Table 4). The majority of the genes in these pathways showed higher expression in fetuses from dams fed DG diet; this pattern is more noticeable in the GO term *dephosphorylation* (Table 4). Moreover, 10 different GO terms that belong to the biological process domain showed significant enrichment (FDR ≤0.05) of differentially expressed genes detected between DG and CN maternal diets (Figure 2A). These functional categories are mainly involved in embryo and fetal development (e.g., *tissue development*, *organ development*, *multicellular organismal development*) and ion transport (e.g., *cation transport*, *calcium ion transport*, *metal ion transport*). Remarkably, the expression of most of the genes in these pathways was increased by maternal DG dietary treatment (Figure 2A).

**Table 4:** **Gene Ontology terms significantly enriched with differentially expressed genes (DEG) between DG and CN or HY maternal diets in fetal subcutaneous adipose tissue**

GO term	DG vs CN			DG vs HY		
	No. genes	No. DEG (↑ DG)	q-value	No. genes	No. DEG (↑ DG)	q-value
Dephosphorylation (0016311)	106	24 (21)	0.025	108	29 (21)	0.035
Phosphatase activity (0016791)	156	29 (18)	0.050	160	36 (21)	0.030
Phosphoric Ester Hydrolase Activity (0042578)	199	36 (25)	0.043	205	46 (27)	0.008

Diets fed to dams: HY = ad libitum fed alfalfa haylage; CN = limit-fed whole shell corn; DG = limit-fed corn dried distiller’s grains.

### Differential gene expression in perirenal adipose tissue

A total of 18,310 genes were evaluated for differential expression between maternal diets in fetal perirenal adipose tissue. Among the genes analyzed, 208, 109 and 27 genes showed differential expression (FDR ≤0.05) between DG vs. CN, DG vs. HY, and CN vs. HY, respectively (Figure 1E). Marked differences were found only between DG and CN maternal diets; none of the other two pairwise comparisons detected significant genes at FDR ≤0.01. Most of the significant genes showed higher expression in fetuses derived from dams fed DG diet.

We further characterized the 208 genes that showed differential expression (FDR ≤0.05) between DG and CN maternal dietary feed sources. Many of these genes are associated with embryo and fetal development [e.g., integrin, beta 4 (*ITGB4*), latent transforming growth factor beta binding protein 4 (*LTBP4*), methyl-CpG binding domain protein 3 (*MBD3*), retinol binding protein 4 (*RBP4*), secreted frizzled-related protein 5 (*SFRP5*)], adipogenesis [e.g., CCAAT/enhancer binding protein (C/EBP), beta (*CEBPB*), delta-like 1 homolog (Drosophila) (*DLK1*), lamin A/C (*LMNA*)], and lipid metabolic process [e.g., acetyl-CoA acyltransferase 1 (*ACAA1*), apolipoprotein D (*APOD*), apolipoprotein E (*APOE*), low density lipoprotein receptor-related protein 1 (*LRP1*), prostaglandin I2 (prostacyclin) synthase (*PTGIS*)]. All these genes were up-regulated in the perirenal adipose tissue of fetuses derived from dams fed the DG diet (FDR ≤0.05).

Gene set enrichment analysis revealed 10 GO terms from the biological process domain significantly enriched with genes differentially expressed between DG and CN maternal diets (Figure 2B). These GO terms are closely related to fetal development (e.g., *tissue development*, *organ development*, *anatomical structure development*), cellular adhesion (e.g., *cell adhesion*, *cell-substrate adhesion*) and Wnt signaling pathway. The expression of most genes in these functional categories was increased by maternal DG diet (Figure 2B). Interestingly, many of these significant GO terms, especially those related to tissue and organ development, were also enriched with significant genes detected between DG and CN maternal diets in fetal subcutaneous fat tissue.

### Validation of overall gene expression

**Figure 3:**
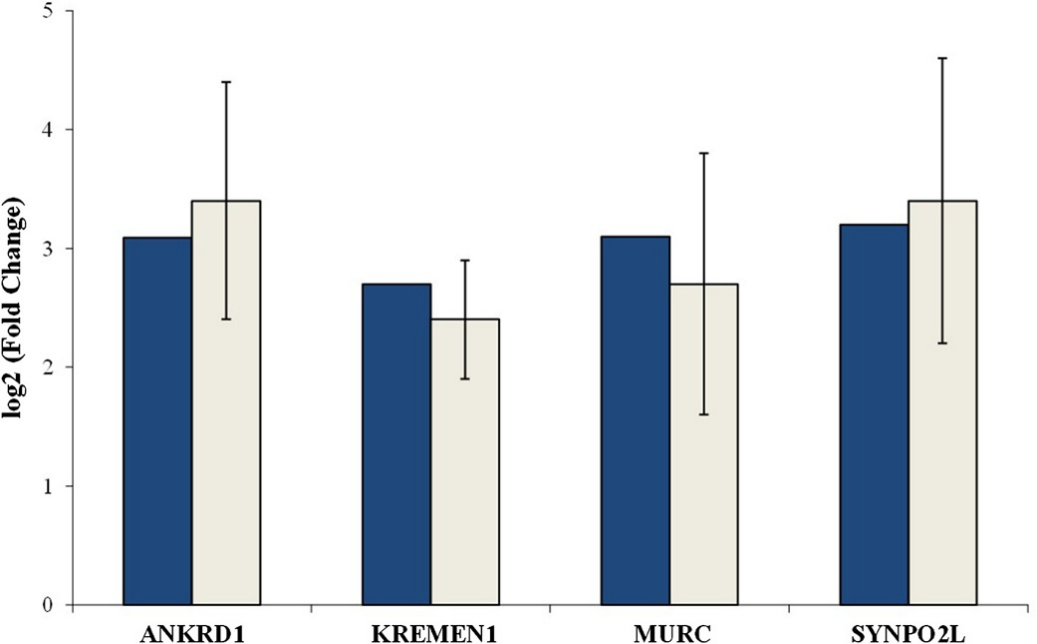
**Fold changes of four differentially expressed genes measured by RNA-Seq (blue) versus qRT-PCR (light grey).** The four genes show higher expression in subcutaneous fat in fetuses from DG diet compared with CN maternal diet.

To validate genes found to be significant in the RNA-Seq analysis, four differentially expressed genes detected in fetal subcutaneous adipose tissue (*ANKRD1*, *KREMEN1*, *MURC*, and *SYNPO2L*) were selected and their expression was assessed using qRT-PCR. RNA-Seq analysis revealed higher expression of these four genes in subcutaneous fat in fetuses derived from DG-fed dams compared with fetuses from CN-fed dams. Figure 3 displays the fold differences in gene expression measured by both RNA-Seq and qRT-PCR. The four genes showed similar patterns of mRNA abundance with both methods (Figure 3).

## Discussion

There is growing evidence that nutritional perturbations during different stages of pregnancy can modify the fetal development of skeletal muscle and adipose tissues, which in turn could have important implications in food animal production [9, 21, 27, 43, 44]. The present study was specifically designed to determine whether maternal diets differing in feed source and nutritive components during mid-to-late gestation impacts the transcriptome of fetal muscle and adipose tissues in sheep. Maternal diets, formulated to provide isoenergetic intakes, differed in the primary feed energy source. More specifically, for ewes fed HY, the energy source was primarily fiber while energy source for ewes fed CN was primarily starch, and for ewes fed DG, energy came from the combination of fiber, fat, and protein thus impacting the substrate supply to the fetus. Additionally, maternal diets differed in protein and fat content. Dams consuming the CN diet had a lower protein intake than dams consuming DG and HY diets, whereas dam consuming the DG diet had higher fat intake than dams consuming CN and HY feedstuffs (see Table 1). Remarkably, these prepartum diets caused notable changes in gene expression in fetal longissimus dorsi muscle, subcutaneous, and perirenal adipose tissues. In particular, maternal CN diet mainly impacted fetal muscle development while the DG diet mainly affected the development of fetal adipose depots. Hence, our findings support the idea that maternal nutrition during mid-to-late gestation can impact the programing of fetal muscle and fat tissues in sheep.

### Maternal nutrition impacts fetal tissue development including myogenesis and adipogenesis

Remarkable gene expression differences in longissimus dorsi muscle were found between fetuses from CN-fed dams and fetuses from either DG-fed or HY-fed dams (Figure 1A-B). Many of the most significantly altered genes are associated with skeletal muscle development. For instance, *MYOD1* encodes a nuclear protein that belongs to the myogenic basic helix-loop-helix transcription factor family, and plays key roles in skeletal muscle cell specification and differentiation [45]. *ANKRD1*, a member of the muscle ankyrin repeat protein family, is a transcription factor involved in the signaling cascade during myogenic differentiation, myofibrillar assembly, and muscle remodeling [46]. *HIRA* is a replication-independent histone chaperone that is linked to transcription and various developmental processes, including the activation of relevant genes during skeletal myogenesis [47]. *BCL9* is an essential component of canonical Wnt signaling cascade that mediates myogenic differentiation during muscle development and regeneration [48]. Importantly, the expression of all these genes was decreased in fetuses derived from dams fed the CN diet. In addition, relevant genes associated with muscle myosin complex and sarcomere organization also showed differential gene expression due to CN maternal diet. For instance, *NRAP* is a muscle-specific scaffolding protein that is involved in myofibril assembly and sarcomere organization [49]. *PARVB* is localized at regions of the sarcolemma in skeletal muscle and plays both physical and signaling roles in muscle cells [50]. *TCAP* is a key component of the Z-disk in striated and cardiac muscle and has a relevant role in sarcomere assembly and integrity [51]. *SYNPO2L* is another component of the Z-disk with important roles in heart and skeletal muscle function and development [52]. These genes were also down-regulated in the muscle of fetuses derived from CN-fed dams. Overall, these results suggest that a prepartum maternal CN diet, i.e., starch-based diet, altered myogenesis, myofibrillogenesis and sarcomere organization in fetal longissimus dorsi muscle. Crude protein intake of ewes fed CN was less than that for ewes fed DG and HY. Interestingly, previous studies have shown that low protein maternal diets, compared against high protein diets fed at isocaloric intakes, adversely affected myogenesis, muscle differentiation, and muscle fiber number in newborn and fattening piglets [53, 54]. Although it seems that a high-starch maternal diet during pregnancy does not affect muscle weights in sheep progeny at birth [31], the possible effect of this diet on morphological and histological parameters of the muscle remains unknown and warrants future studies.

Fetal adipose tissues also showed notable gene expression changes due to different maternal dietary treatments (Figure 1C-F). Among the most significant genes, many are closely related to adipose tissue development. For instance, *ARID5B*, a member of the AT-rich interaction domain family of DNA binding proteins, regulates the transcription of key genes involved in adipogenesis, including adipogenic transcription factors C/EBPα, and PPARγ [55]. *CEBPB*, a member of CCAAT/enhancer binding protein family, encodes a transcription factor whose expression is induced directly by adipogenic hormones, and which plays essential early catalytic roles in the adipocyte differentiation program [56]. *LMNA* encodes an intermediate filament protein localized in the nuclear lamina and has crucial roles in early post-natal differentiation and maturation of adipose tissue [57]. All these genes showed higher expression in fat tissues from fetuses derived from DG-fed dams. In addition, gene set enrichment analysis revealed that GO functional categories related to tissue development and organogenesis, such as *tissue development*, *organ development*, and *multicellular organismal development*, were significantly enriched with differentially expressed genes (Figure 2). These gene expression changes were more pronounced between DG and CN diets, and in general, genes in these GO terms showed higher expression in fetuses derived from DG-fed dams. It is important to note that dams consuming the DG prepartum diet had greater protein and fat intake than dams consuming the CN diet, and hence our findings suggest that prepartum diets with greater fat and protein content may alter fetal adipogenesis and tissue development. Previous studies have investigated the impact that different prepartum diets have on fetal adipose tissues. Larson et al. [17] have shown that maternal protein supplementation during late gestation increased overall percentage of body fat in steer progeny. Similarly, Underwood et al. [58] have shown that a low protein maternal diet negatively impact subcutaneous adipose tissue deposition in beef. In contrast, Rehfeldt et al. [53] reported that either high or low protein maternal diets, fed at isocaloric intakes during pregnancy, reduced both subcutaneous and perirenal fat content in newborn piglets. Furthermore, there is evidence that ingestion of a high-fat diet during pregnancy induced hypertrophy and hyperplasia of fetal adipose tissue [59]. Interestingly, Radunz et al. [15] have shown that a prepartum diet based on dried distillers grains, similar to our DG maternal diet, tended to increase overall internal fat, including perirenal fat, in sheep progeny.

Of particular interest, the term *Wnt receptor signaling pathway* showed an overrepresentation of significant genes in fetal perirenal fat tissue (Figure 2B). The seven genes that revealed differential expression were up-regulated in fetuses from DG-fed dams. Interestingly, these seven genes, *AXIN1*, *DKK3*, *LRP1*, *LRP4*, *SFRP1*, *SFRP2*, and *SFRP4*, are all negative regulators of the Wnt receptor signaling pathway [60, 61]. It is well established that the Wnt receptor signaling pathway inhibits adipogenesis and hence the repression of this pathway is crucial for adipocyte differentiation and fat tissue development [61, 62]. Therefore, these findings provide further evidence of the potential impact of a DG maternal diet on fetal adipose tissue development.

### Maternal nutrition alters metabolic processes in fetal tissues

Prepartum maternal diets impacted the energetic metabolism of fetal muscle. These changes were more marked between fetuses from CN-fed dams vs. HY-fed dams, i.e., starch-based vs. fiber-based maternal diets. Several genes involved in energetic metabolism, including those implicated in *glycolysis and gluconeogenesis*, *citrate cycle*, and *valine, leucine and isoleucine degradation*, were down-regulated by maternal CN diet (Table 3). Similarly, a recent study has shown that a low protein prepartum diet compared against a high protein control diet when fed at isoenergetic intake in mice, caused downregulation of several pathways related to energetic metabolism processes including glycolysis, citrate cycle, and amino acid degradation, in skeletal muscle in newborn offspring [63]. Additionally, fetuses derived from HY-fed dams showed increased expression of genes involved in *propanoate metabolism* and *insulin signaling pathway* (Table 3). Overall, these findings show that prepartum maternal isoenergetic diets, differing in the primary feed source, alter the energetic metabolism of fetal muscle.

Maternal dietary treatments also perturbed different biological and molecular processes in fetal adipose tissues. For instance, in subcutaneous adipose tissue, three functional categories closely related to metabolism of phosphate anhydrides and esters showed overrepresentation of differentially expressed genes (Table 4). GO terms *dephosphorylation*, *phosphatase activity*, and *phosphoric ester hydrolase activity* were mainly enriched with genes that showed significant higher expression in fetuses derived from DG-fed dams. Many relevant biochemical reactions in adipose tissue, including the formation of acyl CoA during the synthesis of fatty acids, involved phosphate anhydrides and esters. Other important processes, such as *ion transport* in subcutaneous fat depot and *cell adhesion* in perirenal fat tissue, were also altered by prepartum maternal diets, showing an up-regulation of gene expression induced by a DG diet (Figure 2). It is well documented that each fat depot has its own particular features, including differences in growth, cellularity and metabolism [64]. This may explain why maternal DG diet altered mainly metabolism processes in subcutaneous fat while it affected mainly cellularity and tissue integrity in perirenal fat depot.

### Potential mechanisms underlying the observed transcriptomic changes

There is growing evidence that maternal nutrition can induce epigenetic alterations to change gene expression in the fetal genome, such as DNA methylation or histone modifications including methylation, acetylation, and phosphorylation [65]. These modifications depend on the availability of key compounds, such as methyl donors, supplied by different amino acids and vitamins present in the maternal diet. Indeed, this link between maternal nutrition and subsequent modification of gene expression in the fetal genome is one of the important molecular mechanisms proposed to explain the phenomenon of fetal programming [65–68]. We have recently reported that maternal DG and HY diets were associated with altered DNA methylation and expression of the imprinted genes *IGF2R* and *H19* in fetal sheep tissues compared to maternal CN diet [32]. Interestingly, while DNA methylation at the gene promoter suppressed expression, gene body methylation was positively associated with gene expression [32]. Here, two functional terms related to chromatin biology and epigenetics, *chromatin* and *nucleosome assembly*, were altered in fetal muscle showing significantly lower expression in fetuses from CN-fed dams. Many genes from these pathways, such as *H1FX*, *HIST2H2BE*, *MBD3*, and *SATB1*, were also differentially expressed in fetal adipose tissues, showing lower expression in fetuses from maternal CN diet. Of special interest is *MBD3*, an essential member of the nucleosome remodeling and deacetylation (NuRD) complex, a multi-functional complex which has key roles in chromatin remodeling during development [69]. *MBD3* is associated with sites in the genome with activated genes, i.e., sites devoid of methylated CpG islands and with chromatin modifications that are characteristic of an active chromatin state [70]. Although DNA methylation or histone modifications were not directly evaluated in our experiment, it seems likely that maternal diets altered chromatin marks of key regions of DNA and these changes probably underlie the transcriptomic changes observed in fetal tissues.

## Conclusions

The present study has characterized the effects of different maternal feed sources during mid-to-late gestation on the transcriptome of fetal tissues in sheep. These prepartum maternal diets caused notable gene expression changes in fetal adipose and muscle tissues. In particular, a maternal starch-based diet mainly altered fetal muscle development while a maternal diet with high fiber, protein, and fat concentrations mainly impacted fetal subcutaneous and perirenal adipose tissues. These findings provide evidence that maternal nutrition during mid-to-late gestation can perturb the programming of fetal muscle and fat tissues in sheep, which in turn may have important implications in food animal production. Determination of the potential effects of these maternal diets on epigenetic modifications in the fetal genome from these tissues is needed in future studies. In sum, the functional ramifications of the observed changes in gene expression, in terms of postnatal growth, body composition, and meat quality of the offspring, also warrant future investigation.

## Electronic supplementary material

Additional file 1: **Description of the pools in terms of number of fetuses and their dams.** (DOCX 14 KB)

Additional file 2: **Summary of sequencing read alignments to the reference genome.** (DOCX 18 KB)
