# Supplementary material for: Maternal nutrition induces gene expression changes in fetal muscle and adipose tissues in sheep
Source: BMC Genomics. 2014 Nov 28;15(1):1034. doi: 10.1186/1471-2164-15-1034 (PMC4301459; doi:10.1186/1471-2164-15-1034)
Supplement: Supplementary file 1 — Additional file 1: Description of the pools in terms of number of fetuses and their dams. (DOCX 14 KB) [file 12864_2014_6846_MOESM1_ESM.docx]

**Additional File 1. Description of the pools in terms of number of fetuses and their dams**

| **Sex** | **CN** | | **HY** | | **DG** | |
| --- | --- | --- | --- | --- | --- | --- |
|  | ***Pool 1*** | ***Pool 2*** | ***Pool 1*** | ***Pool 2*** | ***Pool 1*** | ***Pool 2*** |
| **Female** | 6397 A | 8458 A | 7343 B | 6385 A | 5399 B | 7348 B |
|  | 5393 A |  | 8330 A | 7343 A | 8325 A | 8569 A |
|  | ***Pool 1*** | ***Pool 2*** | ***Pool 1*** | ***Pool 2*** | ***Pool 1*** | ***Pool 2*** |
| **Male** | 8382 C | 5393 B | 8330 B | 6385 B | 5399 A | 7348 A |
|  | 7307 A | 8382 B | 7321 A | 7490 A | 8348 B | 8348 A |
|  | 7307 B | 8382 A | 7321 B |  |  |  |

Diets: **CN** = limit-fed whole shell corn; **HY** = ad libitum fed alfalfa haylage; **DG** = limit-fed corn dried distillers grains. Numbers represent dams and letters represent offspring for a given dam (e.g., A and B means twins from a given dam). A total of 26 fetuses were removed from 15 different dams.
