# Supplementary material for: Maternal nutrition induces gene expression changes in fetal muscle and adipose tissues in sheep
Source: BMC Genomics. 2014 Nov 28;15(1):1034. doi: 10.1186/1471-2164-15-1034 (PMC4301459; doi:10.1186/1471-2164-15-1034)
Supplement: Supplementary file 2 — Additional file 2: Summary of sequencing read alignments to the reference genome. (DOCX 18 KB) [file 12864_2014_6846_MOESM2_ESM.docx]

**Additional File 2. Summary of sequencing read alignments to the reference genome**

**1. Longissimus dorsi muscle tissue**

| **Tissue** | **Diet** | **Sample** | **Total Reads** | **Mapped Reads** | **% Mapped** |
| --- | --- | --- | --- | --- | --- |
| Muscle | CN | F1 | 30,466,627 | 25,282,917 | 83.0 |
| Muscle | CN | F2 | 31,717,064 | 27,022,652 | 85.2 |
| Muscle | CN | M1 | 24,798,496 | 20,982,466 | 84.6 |
| Muscle | CN | M2 | 28,393,680 | 22,361,396 | 78.8 |
| Muscle | DG | F1 | 35,125,090 | 29,278,564 | 83.4 |
| Muscle | DG | F2 | 39,418,267 | `32,758,516 | 83.1 |
| Muscle | DG | M1 | 32,211,325 | 26,681,957 | 82.8 |
| Muscle | DG | M2 | 46,662,154 | 39,892,555 | 85.5 |
| Muscle | HY | F1 | 35,507,864 | 29,588,600 | 83.3 |
| Muscle | HY | F2 | 35,967,553 | 30,557,635 | 85 |
| Muscle | HY | M1 | 32,752,897 | 27,583,039 | 84.2 |
| Muscle | HY | M2 | 31,268,268 | 25,976,151 | 83.1 |

**CN** = limit-fed whole shell corn; **HY** = ad libitum fed alfalfa haylage; **DG** = limit-fed corn dried distillers grains

**2. Subcutaneous adipose depot tissue**

| **Tissue** | **Diet** | **Sample** | **Total Reads** | **Mapped Reads** | **% Mapped** |
| --- | --- | --- | --- | --- | --- |
| Subcutaneous fat | CN | F1 | 35,004,292 | 30,019,366 | 85.8 |
| Subcutaneous fat | CN | F2 | 36,589,297 | 31,794,827 | 86.9 |
| Subcutaneous fat | CN | M1 | 29,224,628 | 24,048,684 | 82.3 |
| Subcutaneous fat | CN | M2 | 23,362,669 | 19,136,134 | 81.9 |
| Subcutaneous fat | DG | F1 | 34,918,074 | 29,719,260 | 85.1 |
| Subcutaneous fat | DG | F2 | 28,745,771 | 25,468,326 | 88.6 |
| Subcutaneous fat | DG | M1 | 25,297,981 | 20,601,607 | 81.4 |
| Subcutaneous fat | DG | M2 | 30,724,288 | 24,896,029 | 81.0 |
| Subcutaneous fat | HY | F1 | 30,005,651 | 22,573,636 | 75.2 |
| Subcutaneous fat | HY | F2 | 35,169,747 | 29,708,215 | 84.5 |
| Subcutaneous fat | HY | M1 | 37,031,027 | 30,017,133 | 81.1 |
| Subcutaneous fat | HY | M2 | 33,120,661 | 27,596,913 | 83.3 |

**CN** = limit-fed whole shell corn; **HY** = ad libitum fed alfalfa haylage; **DG** = limit-fed corn dried distillers grains

**3. Perirenal adipose depot tissue**

| **Tissue** | **Diet** | **Sample** | **Total Reads** | **Mapped Reads** | **% Mapped** |
| --- | --- | --- | --- | --- | --- |
| Perirenal Fat | CN | F1 | 37,998,816 | 31,745,141 | 83.5 |
| Perirenal Fat | CN | F2 | 36,354,523 | 30,398,882 | 83.6 |
| Perirenal Fat | CN | M1 | 32,483,262 | 26,973,062 | 83.0 |
| Perirenal Fat | CN | M2 | 34,939,612 | 29,907,057 | 85.6 |
| Perirenal Fat | DG | F1 | 34,980,968 | 30,693,978 | 87.7 |
| Perirenal Fat | DG | F2 | 29,048,137 | 24,699,070 | 85.0 |
| Perirenal Fat | DG | M1 | 28,302,684 | 23,792,194 | 84.1 |
| Perirenal Fat | DG | M2 | 36,750,270 | 30,769,811 | 83.7 |
| Perirenal Fat | HY | F1 | 30,570,877 | 26,901,241 | 88.0 |
| Perirenal Fat | HY | F2 | 27,606,470 | 23,819,576 | 86.3 |
| Perirenal Fat | HY | M1 | 36,592,206 | 31,910,603 | 87.2 |
| Perirenal Fat | HY | M2 | 31,322,311 | 25,827,367 | 82.5 |

**CN** = limit-fed whole shell corn; **HY** = ad libitum fed alfalfa haylage; **DG** = limit-fed corn dried distillers grains
